# Resistome Analysis of a Carbapenemase (OXA-48)-Producing and Colistin-Resistant Klebsiella pneumoniae Strain

**DOI:** 10.1128/AAC.00076-18

**Published:** 2018-04-26

**Authors:** Trestan Pillonel, Patrice Nordmann, Claire Bertelli, Guy Prod' hom, Laurent Poirel, Gilbert Greub

**Affiliations:** a Institute of Microbiology, University of Lausanne and University Hospital Center, Lausanne, Switzerland; b Emerging Antibiotic Resistance Unit, Medical and Molecular Microbiology, Department of Medicine, University of Fribourg, Fribourg, Switzerland; c INSERM European Unit (IAME, France), University of Fribourg, Fribourg, Switzerland; d Swiss National Reference Center for Emerging Antibiotic Resistance (NARA), University of Fribourg, Fribourg, Switzerland

**Keywords:** Klebsiella pneumoniae, carbapenemase, ESBL, genome sequence, polymyxins, ST15, antibiotic resistance, yersiniabactin

## LETTER

Carbapenemase-producing Klebsiella pneumoniae strains are increasingly reported worldwide (1, 2). Therefore, polymyxins (colistin, polymyxin B) often constitute last-resort antibiotics to treat infections due to those multidrug-resistant carbapenemase producers. Here, the genetic basis of the antibiotic resistance determinants of a carbapenem- and colistin-resistant K. pneumoniae isolate was investigated.

A patient was hospitalized at the Bicêtre Hospital (Paris, France) for multiple bone fractures that occurred following a 6-floor fall in Bucharest, Romania. K. pneumoniae FR-1 was recovered from routine rectal screening. This first isolate was resistant to carbapenems, fluoroquinolones, rifampin, trimethoprim-sulfamethoxazole, and fosfomycin. FR-1 produced the carbapenemase OXA-48 and the extended-spectrum β-lactamase CTX-M-15, as determined by two specific PCRs. Then, the patient developed high-grade fever. Since an infection due to resistant Gram-negative bacteria was suspected, he received an empirical antibiotic treatment consisting of colistin (5 mg/kg of body weight/day) and amikacin (15 mg/kg/day) for 2 days. Eight days later, he developed a wound infection from which K. pneumoniae strain FR-2 (with the same resistance profile as FR-1) was recovered. The treatment was therefore switched to tigecycline (100 mg twice/day), colistin (5 mg/kg/day), and amikacin (15 mg/kg/day). Then, another K. pneumoniae isolate (FR-3) was recovered from the same wound and exhibited additional resistance to colistin. The antibiotherapy was modified for doripenem (1 g 4 times/day), fosfomycin (3 g/day), and tigecycline (100 mg twice/day), which cured the infection.

**FIG 1:**
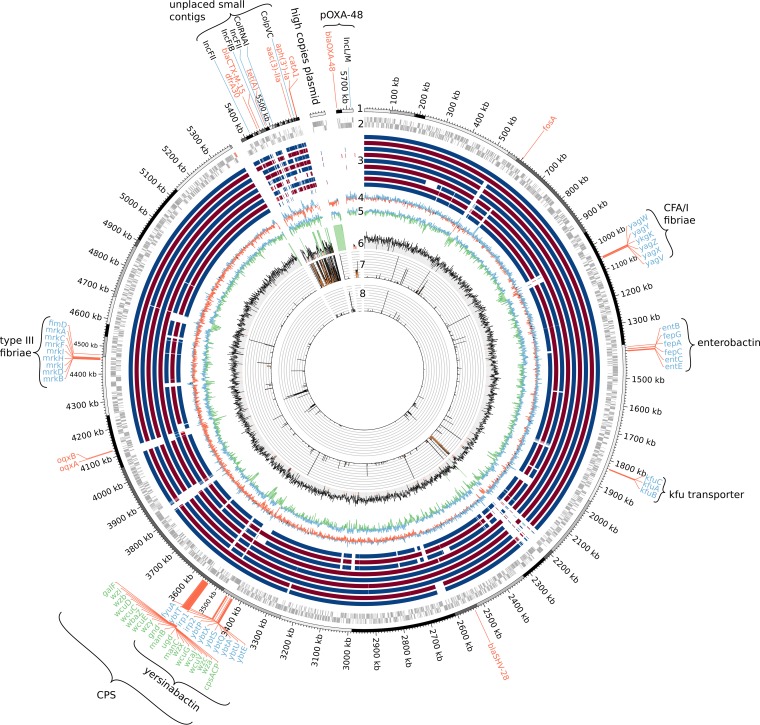
Genome map of the K. pneumoniae FR-3 isolate. Circles are as follows: the first indicates contig boundaries and the second open reading frames borne on the leading and lagging strands. rRNA and tRNA are red. The third circle indicates whole-genome alignments with the following K. pneumoniae strains: two ST15 strains from the Netherlands outbreak (GCF_001596925.1, GCF_001597245.1), two ST15 strains from Nepal (GCF_000764615.1, GCF_000943095.1), two additional ST15 from Taiwan and China (GCF_001750805.1, GCF_001663195.1), and three unrelated ST258, ST11, and ST147 strains (GCF_000598005.1, GCF_000240185.1, GCF_001746535.1, respectively). The fourth circle indicates the GC skew. The fifth indicates GC content, and the sixth is a histogram of the sequencing depth. Regions presenting more than 3-times-higher depth than the median of the assembly are highlighted in green. Regions presenting a sequencing depth lower than half of the median depth are red. The 7th circle is a histogram of the count of significant blastp hits versus those in the RefSeq plasmid database (limited to a maximum of 50). The eighth circle is a histogram of the number of significant blastp hits in the PHAST database. Outer labels highlight relevant genes or operons. Antibiotic resistance genes are red, virulence genes are blue, and probes of known plasmids identified using PlasmidFinder are black.

Genome sequencing (see Methods in the supplemental material) of FR-3 shows that it belongs to sequence type 15 (ST15), a widely distributed multidrug-resistant clone (3, 4). Genomic investigations of two outbreaks involving ST15 clones in Nepal and in the Netherlands in 2012 subdivided this clade in two main lineages harboring distinct capsule synthesis (*cps*) loci (5). FR-3 exhibits the *cps* locus serotype K24, the same serotype as the CTX-M-15-producing outbreak strains from the Netherlands (5). FR-3 and the six ST15 strains from China, Nepal, the Netherlands, and Taiwan compared here harbor the Yersinia high-pathogenicity island and the ferric-uptake operon *kfuABC*, both considered as K. pneumoniae virulence factors (6) (Fig. 1).

Eleven antimicrobial resistance genes were identified (7), in accordance with the phenotypic resistance pattern (Table 1; Table S1). Detailed analysis of the genome identified neither genes encoding ADP-ribosylation (Arr) enzymes nor RpoB polymorphism(s) that could explain the observed resistance to rifampin. PlasmidFinder (8) identified five putative distinct plasmid replicons, namely, ColRNAI, IncL, ColpVC, and IncFIB, and two distinct IncFII replicons with 95.9% identical RNAI-FII sequences. One of the IncFII replicons was identified on the same contig as a resistance gene (*tetA*). The *bla*_OXA-48_ gene was identified on an IncL backbone. The latter plasmid sequence was 99% identical to the previously reported 62-kb sequence of pOXA-48a, known to be self-conjugative and conjugating at high frequency (9).

**TABLE 1:** List of coding sequences associated with drug resistance from the colistin-resistant strain K. pneumoniae FR-3

Gene or protein	Identity to best hit (%) or amino acid change(s)	Best-hit GenBank accession no.	Antibiotic resistance	Contig size (bp)	Depth ratio of gene^*a*^	Depth ratio of contig^*b*^
Genes						
*aac(3)-IIa*	99.77	X51534	Aminoglycosides	2,915	0.56	0.63
*aph(3*′*)-Ia*	100	V00359	Aminoglycosides	1,334	0. 8	0.68
*bla*_SHV-28_^*c*^	100	HM751101	Narrow-spectrum β-lactamase	85,380	0.56	0.8
*bla*_OXA-48_	100	AY236073	Carbapenems	2,231	0.51	0.57
*bla*_CTX-M-15_	100	DQ302097	Extended-spectrum cephalosporins	10,416	0.61	0.63
*oqxA*^*c*^	99.23	EU370913	Quinolones	194,368	0.78	1.02
*oqxB*^*c*^	98.86	EU370913	Quinolones	194,368	1.02	1.02
*fosA*^*c*^	97.62	ACWO01000079	Fosfomycin	362,468	0.73	1.09
*catA1*	99.85	V00622	Phenicols	1,204	0.95	0.83
*tet(A)*	100	AJ517790	Tetracycline	13,075	0.64	0.61
*dfrA30*	99.58	AM997279	Trimethoprim	10,416	0.71	0.63
Proteins						
GyrA^*c*^	S83F, D87A		Quinolones	579,439	1.12	0.99
ParC^*c*^	S80I		Quinolones	103,616	1.14	1.07
MgrB^*c*^	N42Y, K43I		Colistin	227,842	1.04	0.98

a Ratio of the median sequencing depth of the gene to the median sequencing depth of the whole assembly (*n* = 259).

b Ratio of the median sequencing depth of the contig bearing the gene to the median sequencing depth of the whole assembly (*n* = 259).

c Borne or encoded by the chromosome.

All contigs carrying resistance genes exhibited much lower median sequencing depth than the rest of the assembly (between 57 and 83%) (Table 1), similarly to most regions presenting a high number of hits against the RefSeq plasmid database (Fig. 1). This suggests that these genes are located on plasmids. Homologs of type IV secretion system (type F) proteins were identified on several small contigs, indicating that FR-3 may carry a second conjugative plasmid.

Two nonsynonymous mutations previously reported to occur in colistin-resistant strains (10) were identified in the PhoQ/PhoP regulator *mgrB* gene (N42Y, K43I) likely explaining the acquired resistance to colistin. Indeed, substitutions or deletions in the *mgrB* gene of K. pneumoniae are the most frequent molecular mechanisms of acquired resistance to colistin (11). Nonsynonymous mutations were also identified in the *gyrA* (S83F, D87A) and *parC* (S80I) genes and are likely responsible for the acquired resistance to fluoroquinolones (Table 1) (12). Efflux pumps, such as *oqxA* and *oqxB*, might also be involved in the observed resistances.

This analysis characterizes the resistance determinants that have accumulated over time in the FR strain, leading to an almost pan-resistant strain.

## 

### Accession number(s).

The sequences were submitted to ENA under the accession number PRJEB20782.

## Supplementary Material

Supplemental material
