# Supplementary material for: Resistome Analysis of a Carbapenemase (OXA-48)-Producing and Colistin-Resistant Klebsiella pneumoniae Strain
Source: Antimicrob Agents Chemother. 2018 Apr 26;62(5):e00076-18. doi: 10.1128/AAC.00076-18 (PMC5923095; doi:10.1128/AAC.00076-18)
Supplement: Supplemental material [file supp_62_5_e00076-18__index.html]

Supplemental material 

# Resistome Analysis of a Carbapenemase (OXA-48)-Producing and Colistin-Resistant Klebsiella pneumoniae Strain

## Supplemental material

- Supplemental file 1 -

  Supplemental methods and Table S1

  PDF, 80K
