# Supplementary material for: Resistome Analysis of a Carbapenemase (OXA-48)-Producing and Colistin-Resistant Klebsiella pneumoniae Strain
Source: Antimicrob Agents Chemother. 2018 Apr 26;62(5):e00076-18. doi: 10.1128/AAC.00076-18 (PMC5923095; doi:10.1128/AAC.00076-18)
Supplement: Supplemental material [file AAC.00076-18_zac005187113s1.pdf]

Supplementary data: Resistome analysis of a carbapenemase (OXA-48)-producing and colistin-resistant *Klebsiella pneumoniae* strain

Trestan Pillonel[1], Patrice Nordmann[1,2,3,4], Claire Bertelli[1], Guy Prod' hom[1], Laurent Poirel[2,3,4], and Gilbert Greub[1]

[1]Institute of Microbiology, University of Lausanne and University Hospital Center, 48 rue du Bugnon, CH-1011 Lausanne, Switzerland
[2]Emerging Antibiotic Resistance Unit, Medical and Molecular Microbiology, Department of Medecine, University of Fribourg, Fribourg, Switzerland
[3]INSERM European Unit (IAME, France), University of Fribourg, Fribourg, Switzerland
[4]Swiss National Reference Center for Emerging Antibiotic Resistance (NARA), University of Fribourg, Fribourg, Switzerland

January 2018

1 Supplementary methods

Genomic DNA of *K. pneumoniae* was extracted using the Gentra Puregene kit (Qiagen, Hilden, Germany). A genomic DNA library was prepared using Nextera DNA Sample Preparation Kit, tagged using Nextera Index kit (Illumina, San Diego CA), and sequenced on a MiSeq sequencer (Illumina, San Diego CA) following the manufacturer's protocol. Paired-end 250 bp reads were filtered using fastq-mcf [1], keeping reads with an average Phred quality score higher than 30 (Parameters: –max-ns 0 –mean-qual 30 -q 30). The genome was assembled using SPAdes version v3.9.0 with kmer sizes ranging from 27 to 127, by steps of 4 [2]. Contigs were reordered based on the reference genome of *K. pneumoniae* strain HS11286 (accession: NC_016845) using Mauve version 2015.02.13 [4]. Whole-genome alignments were performed with nucmer [6]. The genome was annotated using Prokka version 1.11 [7]. Circos v0.69-4 was used to make a circular genome representations of the genome [5]. The presence of plasmids was investigated using PlasmidFinder [3]. Antimicrobial resistance genes were identified using ResFinder 2.1 [8].

Table S1: Antibiotic susceptibility of *K. pneumoniae* FR-3[1].

Class	Name	Kirby-Bauer [mm]	MIC Vitek [$\mu g/ml$]	MIC Etest [$\mu g/ml$]	Interpretation
Aminopenicillin /	Amoxicillin / Clavulanic Acid	6	>=32	n/a	R
inhibitor combination	Ampicillin	6	>=32	n/a	R
	Ampicillin / Sulbactam	n/a	n/a	>256	R
Beta-lactam/beta-lactamase inhibitor	Piperacilllin / Tazobactam	6	>=128	n/a	R
	Ticarcillin / Clavulanic Acid	n/a	n/a	>256	R
Cephalosporin II /	Cefoxitin	6	>=64	n/a	R
Cephamycin	Cefuroxime	n/a	n/a	>256	R
Cephalosporin III / IV	Cefepime	6	>=64	n/a	R
	Cefpodoxime	6	>=8	n/a	R
	Ceftazidime	6	>=64	n/a	R
	Ceftriaxone	6	>=64	n/a	R
Monobactam	Aztreonam	6	n/a	n/a	R
Carbapenem	Imipenem	13	8	4	R
	Meropenem	6	>=16	16	R
	Ertapenem	6	>=8	>32	R
Aminoglycoside	Amikacin	22	<=2	n/a	S
	Gentamicin	6	n/a	n/a	R
	Tobramycin	12	8	n/a	R
Fluroquinolone	Ciprofloxacin	6	>=4	n/a	R
	Levofloxacin	n/a	n/a	>32	R
	Moxifloxacin	n/a	n/a	8	R
Tetracyclines	Tetracycline	n/a	n/a	>256	R
	Tigecycline	n/a	n/a	2	R
Miscellaneous	Nitrofurantoin	10	128	n/a	R
	Trimethoprim / Sulfamethoxazole	6	>=320	n/a	R
	Rifampicin	n/a	n/a	>32	R
	Chloramphenicol	n/a	n/a	>256	R
	Fosfomycin	n/a	64	96	R
	Colistin	n/a	>=16	16	R

[1] Disk diffusion and gradient tests were determined with cation-adjusted MH broth (Bio-Rad, Cressier, Switzerland). Disks and Etests were obtained from Oxoid, Thermofisher Scientific (Pratteln, Switzerland) and bioMerieux (Marcy-l'Etoile, France) respectively. The minimum inhibitory concentration (MIC) values were determined using the AST-N242 card from VITEK2 (bioMerieux). For colistin, the MIC was determined using UMIC (Biocentric, Bandol, France) according to the manufacturer's instructions. EUCAST standards (version 2017) were used for categorical interpretation.

References

[1] Erik Aronesty. ea-utils: Command-line tools for processing biological sequencing data. *Durham, NC: Expression Analysis*, 2011.

[2] Anton Bankevich, Sergey Nurk, Dmitry Antipov, Alexey A Gurevich, Mikhail Dvorkin, Alexander S Kulikov, Valery M Lesin, Sergey I Nikolenko, Son Pham, Andrey D Prjibelski, et al. Spades: a new genome assembly algorithm and its applications to single-cell sequencing. *Journal of computational biology*, 19(5):455–477, 2012.

[3] Alessandra Carattoli, Ea Zankari, Aurora García-Fernández, Mette Voldby Larsen, Ole Lund, Laura Villa, Frank Møller Aarestrup, and Henrik Hasman. In silico detection and typing of plasmids using plasmidfinder and plasmid multilocus sequence typing. *Antimicrobial agents and chemotherapy*, 58(7):3895–3903, 2014.

[4] Aaron CE Darling, Bob Mau, Frederick R Blattner, and Nicole T Perna. Mauve: multiple alignment of conserved genomic sequence with rearrangements. *Genome research*, 14(7):1394–1403, 2004.

[5] Martin Krzywinski, Jacqueline Schein, Inanc Birol, Joseph Connors, Randy Gascoyne, Doug Horsman, Steven J Jones, and Marco A Marra. Circos: an information aesthetic for comparative genomics. *Genome research*, 19(9):1639–1645, 2009.

[6] Stefan Kurtz, Adam Phillippy, Arthur L Delcher, Michael Smoot, Martin Shumway, Corina Antonescu, and Steven L Salzberg. Versatile and open software for comparing large genomes. *Genome biology*, 5(2):R12, 2004.

[7] Torsten Seemann. Prokka: rapid prokaryotic genome annotation. *Bioinformatics*, 30(14):2068–2069, 2014.

[8] Ea Zankari, Henrik Hasman, Salvatore Cosentino, Martin Vestergaard, Simon Rasmussen, Ole Lund, Frank M Aarestrup, and Mette Voldby Larsen. Identification of acquired antimicrobial resistance genes. *Journal of antimicrobial chemotherapy*, 67(11):2640–2644, 2012.
